# The generation and function of soluble apoE receptors in the CNS

**DOI:** 10.1186/1750-1326-1-15

**Published:** 2006-10-24

**Authors:** G William Rebeck, Mary Jo LaDu, Steven Estus, Guojun Bu, Edwin J Weeber

**Affiliations:** 1 Department of Neuroscience, Georgetown University, Washington DC, USA; 2 Department of Anatomy and Cell Biology, University of Illinois at Chicago, Chicago, USA; 3 Department of Physiology, University of Kentucky, Lexington, USA; 4 Sanders-Brown Center on Aging, University of Kentucky, Lexington, USA; 5 Department of Pediatrics, Washington University, St. Louis, USA; 6 Department of Cell Biology and Physiology, Washington University, St. Louis, USA; 7 Hope Center for Neurological Disorders, Washington University, St. Louis, USA; 8 Department of Molecular Physiology and Biophysics, Vanderbilt University, Nashville, USA; 9 Department of Pharmacology, Vanderbilt University, Nashville, USA; 10 Vanderbilt Kennedy Center for Research on Human Development, Vanderbilt University, Nashville, USA

## Abstract

More than a decade has passed since apolipoprotein E4 (*APOE-ε4*) was identified as a primary risk factor for Alzheimer 's disease (AD), yet researchers are even now struggling to understand how the apolipoprotein system integrates into the puzzle of AD etiology. The specific pathological actions of apoE4, methods of modulating apolipoprotein E4-associated risk, and possible roles of apoE in normal synaptic function are still being debated. These critical questions will never be fully answered without a complete understanding of the life cycle of the apolipoprotein receptors that mediate the uptake, signaling, and degradation of apoE. The present review will focus on apoE receptors as modulators of apoE actions and, in particular, explore the functions of soluble apoE receptors, a field almost entirely overlooked until now.

## Background

### ApoE and apoE receptors

Apolipoprotein E (apoE) is a small (34-kDa) secreted glycoprotein that associates with lipoproteins and mediates uptake of these particles into target cells via receptor-mediated endocytosis by the low density lipoprotein (LDL) receptor family. Three commonly occurring isoforms have been identified in the human population due to single nucleotide polymorphisms on the *APOE *gene on chromosome 19. The apoE3 isoform (Cys112, Arg158) occurs at the highest frequency, followed by apoE4 (Arg112, Arg158) and apoE2 (Cys112, Cys158). ApoE is a ligand for the seven identified mammalian members of the evolutionarily conserved low density lipoprotein (LDL) receptor family: the low density lipoprotein receptor (LDLR), apoE receptor 2 (ApoER2), the very low density lipoprotein receptor (VLDLR), multiple epidermal growth factor (EGF) repeat-containing protein (MEGF7), megalin, LDL-related protein-1 (LRP1) and LDL-related protein-1b (LRP1b). *APOE *was initially recognized for its importance in lipoprotein metabolism and cardiovascular disease, however, more recently it has been studied for its role in several biological processes not directly related to lipoprotein transport. The following review will focus on the role of apoE and apoE receptors in the CNS with a focus on the processing and production of soluble apoE receptors.

### Genetics of apoE and Alzheimer's disease

Alzheimer's disease (AD) is a complex neurodegenerative condition characterized neuropathologically by the presence of extracellular amyloid plaques, intraneuronal neurofibrillary tangles, and neuronal loss. While most AD is sporadic in nature, two classes of genetic risk factors for AD have been identified. The first class consists of mutations responsible for the rare, familial AD (FAD). Genes implicated in FAD include the β-amyloid protein precursor (APP), presenilin-1, and presenilin-2; mutations in these genes produce AD risk marked by autosomal dominance, early disease onset, high penetrance, and relative rarity, on the order of hundreds of families worldwide (reviewed in [1]). Each of the mutated forms of these genes enhances the production of amyloid-β (Aβ) peptide [2], particularly the 42 amino acid form (Aβ42). Because Aβ42 aggregates to form amyloid, the discovery of these genes strongly supports the "amyloid hypothesis" [2].

A second class of AD risk factor is genetic variation that modulates the sporadic late onset AD; at present, the sole member of this class is the *APOE *gene. *APOE *encodes a secreted protein of 299 amino acids important for the transport of cholesterol. Single nucleotide polymorphisms (SNP) define three common alleles (ε2, ε3, and ε4), which encode proteins that differ at two amino acids. *APOE-ε2 *is associated with reduced odds and delayed onset of AD while *APOE-ε4 *is associated with increased odds and earlier onset of AD [3]. *APOE-ε4 *appears to account for up to 40–50% of the genetic risk of AD [4,5]. Furthermore, *APOE-ε4 *is associated with increased brain Aβ in affected individuals [6]. The effects of *APOE *on Aβ burden are seen in mice as well, with studies in mice deficient for apoE or transgenic for human apoE supporting a role for apoE in Aβ fibrillogenesis and neuritic plaque formation [7,8]. Importantly, the risk associated with *APOE-ε4 *is modulated by other unknown genetic and environmental factors.

AD has a complex etiology that encompasses environmental factors as well as the genetic risk factors. The existence of environmental risk factors is demonstrated by multiple lines of evidence, including studies of twins. Interestingly, it was found that only about half of identical twins have concordance for AD [9,10]. Among the environmental factors identified, cholesterol lowering treatments (reviewed in [11,12]) and anti-inflammatory drugs (reviewed in [13]) may decrease risk of AD. Alternatively, analysis of post-mortem AD brains showed an increased level of traumatic brain injury compared to normal non-AD brains, suggesting that brain trauma may significantly increase the risk of AD [14]. ApoE is potentially involved in each of these environmental factors.

### ApoE and apoE receptors in the CNS

#### ApoE and cholesterol transport in the CNS

In the periphery, there are numerous lipoprotein classes and apolipoproteins. However, in the CNS, lipoproteins are predominantly high density and do not include the large classes of lower density lipoproteins found in the plasma [15]. The two major apolipoproteins present in the cerebral spinal fluid (CSF) are apoE and apoAI; classes of high density lipoproteins in the CSF contain either or both of these apolipoproteins [16]. These lipoproteins can act either to deliver cholesterol to cells, or to remove excess cholesterol from cells [17]. The additional role of these proteins as signaling molecules will be discussed later in this review.

#### ApoE and Aβ

The strongest associations of *APOE *genotype with disease are with conditions containing amyloid deposition including AD, Down's syndrome, cerebral amyloid angiopathy and head trauma [6,18-23]. *In vivo*, early evidence for an involvement of apoE in AD came from immunohistochemical localization of apoE to senile plaques [24]. ApoE4 increases levels of amyloid deposition in humans [6,18] and accelerates amyloid deposition in transgenic mice [8,25,26]. Recent research has focused on soluble oligomeric assemblies of Aβ as the proximate cause of neuronal injury, synaptic loss and the eventual dementia associated with AD [27-31]. ApoE binds soluble Aβ oligomers found in the brain, plasma and CSF [32,33]. In vitro, apoE forms stable complexes with Aβ [34-37], alters the aggregation of various Aβ peptides [38-40], modulates Aβ-induced neuroinflammation [41-44], and promotes Aβ clearance [45]. In addition, Aβ42 and apoE4 act synergistically to reduce neuronal viability *in vitro* and ex vivo as measured by neurotoxicity in primary cultures and impaired long term potentiation (LTP) in hippocampal slice cultures [46-51]. Each of these important functions is partially mediated by apoE receptors.

#### ApoE receptors

**Figure 1:**
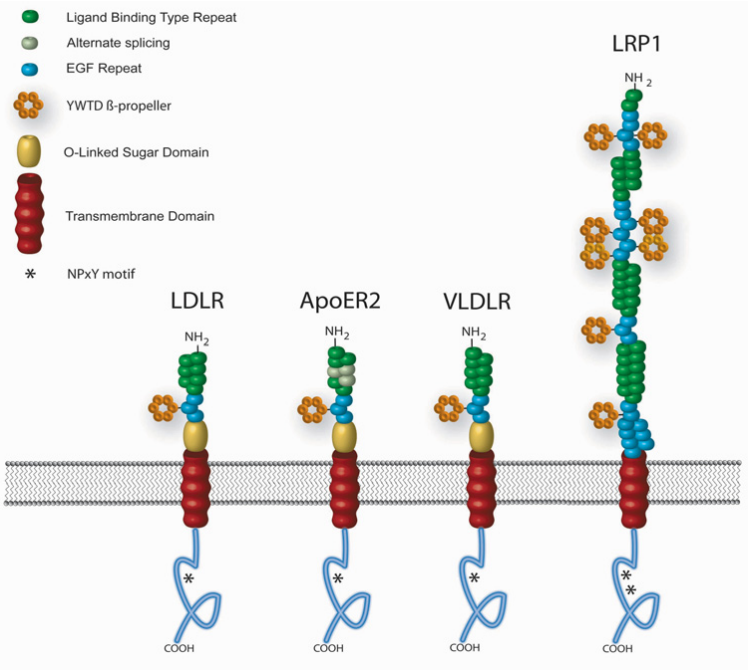
**LDL receptor family members**. LDLR, ApoER2, VLDLR and LRP represent the four major apoE receptor family members in the mammalian CNS. Each member contains a single transmembrane domain, at least one ligand binding domain, EGF repeat, YWTD β-propeller and cytoplasmic NPxY motif.

ApoE interacts with members of the LDL receptor family on the surface of cells. The LDLR family consists of over ten receptors that function in receptor-mediated endocytosis and cellular signaling (Figure 1) [52,53]. In addition to the LDLR itself, the family includes LRP/LRP1 [54], megalin/LRP2 [55], VLDLR [56], ApoER2/LRP8 [57-59], SORLA-1/LR11 [60,61], LRP4 [62], LRP5 [63,64], LRP6 [65], and LRP1B [66]. The most characteristic structural component of the LDLR family is the cysteine-rich ligand-binding repeats forming ligand-binding domains [52,67]. Additionally, most members of the LDLR family contain epidermal growth factor (EGF)-like repeats and YWTD motifs, which form β-propeller-like structures [68]. A common feature that is shared by most members of the LDLR family is their ability to bind the receptor-associated protein (RAP) [69]. RAP is an endoplasmic reticulum (ER)-resident protein that functions in receptor folding and trafficking along the early secretory pathway and universally antagonizes ligand-binding to all members of the family [69].

Many of the apoE receptors have been found in the CNS. Neurons express LDLR, LRP, ApoER2, and the VLDLR; astrocytes express LDLR and LRP; microglia express VLDLR and LRP [6,57,70-73]. It is unclear which receptors are expressed on oligodendrocytes. Soluble forms of each of these receptors have been detected (see below).

#### ApoE receptors and endocytosis

A major function of at least some of the apoE receptors is clathrin-mediated endocytosis. The rapid endocytosis rate of LRP is unique among LDLR family members. The dominant endocytosis signal for LRP is the Y*XX*L motif [74]. The two copies of the NP*X*Y motif, which mediates LDLR endocytosis [75], do not play significant roles in LRP endocytosis but likely bridge interactions with adaptor proteins for signaling and intracellular trafficking. The initial endocytosis rates of individual LDLR family members are significantly different with half time for internalization ranging from <0.5 min (LRP) to > 8 min (ApoER2/VLDLR) ([76], see Table 1). These results suggest endocytic functions among LDLR family members are distinct. In addition to endocytosis, LDLR family members also exhibit efficient recycling. In particular, sorting nexin 17, a member of the PX-domain containing, sorting nexin family, interacts with the proximal NPXY motif of the LRP tail and promotes its recycling in the early endosome [77]. Other adaptor proteins, specifically Dab-1 and FE65, affect levels of surface apoE receptors [78]. Although significant cholesterol from the periphery does not get transported into the CNS and sufficient cholesterol is synthesized in the CNS [79], cholesterol redistribution is important for transport of cholesterol from glia to neurons [80] and for clearance of membrane debris after CNS damages [81].

**Table 1:** ApoE receptor endocytosis rates.

Receptor	t_1/2_
LRP	0.5 min
LDLR	4.8 min
ApoER2	8.1 min
VLDLR	8.2 min

#### ApoE receptors and intracellular signaling

Recently, several studies have demonstrated a role for some apoE receptors (specifically LRP, ApoER2, and VLDLR) as signaling molecules (see [53,82] for review). VLDLR and ApoER2 transduce signals from the extracellular matrix molecule Reelin, affecting neuronal cell migration during development [83]. LRP activation by ligand binding affects NMDA receptor function [84-89]. These effects are transduced through the cytoplasmic domains of receptors binding various cytoplasmic adaptor and scaffold proteins containing PID or PDZ domains, including mammalian Disabled-1 (mDab1), mDab2, FE65, JNK-interacting protein JIP-1 and JIP-2, and PSD-95 [90-95]. ApoE receptor ligands also promote other intraneuronal signals via apoE receptors, including PI3 activation, ERK activation, and JNK inhibition [86,96,97] but exactly which receptors promote which signals is unknown. ApoE receptors on glia also affect signaling pathways, affecting the state of glial activation [41,42,98].

#### ApoE receptors and synaptic plasticity

These receptor-mediated processes defined *in vitro* are important for brain physiological functions. The apoE receptor antagonist RAP prevents induction of long-term potentiation (LTP) in hippocampal slices [69]. ApoER2 and VLDLR knock-out (KO) mice have normal baseline synaptic transmission, as measured in acute hippocampal slices, but have subtle impairment of hippocampal LTP [83,99]. Moreover, Reelin application enhanced LTP induction, which was dependent on the presence of both ApoER2 and VLDLR [97]. A potential molecular mechanism for this function of ApoER2 is a 59 amino acid cytoplasmic domain that is alternatively spliced. This ApoER2 splice variant interacts with PSD-95, which is itself associated with NMDA receptor conductance [87,95]. Knock-in mice exclusively expressing ApoER2 receptors that lack the 59 amino acid insert exhibit decreased LTP induction, and no enhancement of LTP in the presence of exogenous Reelin [87]. Thus, the role of ApoER2 in LTP appears to be in the capacity of NMDA receptor modulation by increasing NMDAR conductance and thus indirectly altering intracellular calcium levels.

#### ApoE isoforms and synaptic plasticity

Increasing evidence indicates that apoE4 itself impairs neuronal viability. Not all cell types are susceptible to apoE4-induced toxicity; glia are relatively resistant [100] and only cells with a neuronal phenotype appear vulnerable [101]. ApoE4 actually inhibits neurite outgrowth, overrides the neurite-stimulatory effect of apoE3 and is neurotoxic *in vitro *(reviewed by Teter [102]). Transgenic expression of human apoE4 has dominant negative behavioral effects [103-106], including deficits in memory tasks [103,105]. In addition, apoE4 mice a exhibit greater memory impairment than apoE-knockout (apoE-KO) mice, suggesting that apoE4 confers a gain of negative function [106,107].

Several studies utilizing genetically altered mice have begun to shed light on the roles of apoE in synaptic plasticity and memory formation. ApoE targeted replacement mice (apoE-TR mice) express one of the three human isoforms under the control of endogenous murine *APOE* promoter sequences in a conformation and at physiological levels in a temporal and spatial pattern comparable to endogenous mouse apoE [108]. ApoE-TR mice expressing the apoE3 isoform are identical to wild type mice in both LTP induction and spatial learning. In contrast, mice expressing the apoE4 isoform demonstrate compromised LTP induction and spatial learning. Importantly, the impaired spatial learning exhibited by apoE-deficient mice can be rescued by infusion of human apoE3 or apoE4 [109,110]. Thus, apoE and its receptors influence NMDA receptor activity, LTP, and spatial memory.

#### ApoE, Aβ, and synaptic plasticity

In addressing the effect of apoE on Aβ-induced changes in neuronal viability, it is unclear precisely what form of the Aβ peptide was used in early studies because it has been difficult to isolate and determine the conformational species of Aβ responsible for its neural activity [50,51]. *In vitro*, several recent studies have demonstrated that apoE2 and E3, but not E4, protect neurons against cell death induced by non-fibrillar Aβ, but have no effect on fibrillar-induced toxicity [111,112]. In addition, oligomeric Aβ42-induced neurotoxicity is significantly greater in both Neuro-2A cells treated with apoE4 [112-114] and primary co-cultures of wild-type (WT) neurons and glia from apoE-TR mice expressing apoE4 [47,112]. Using apoE-TR mice, oligomeric Aβ42-induced inhibition of LTP was greatest in hippocampal slice cultures from apoE4-TR mice, while apoE2 actually protected against LTP impairment [46]. *In vivo*, crossing apoE2 transgenic mice with APP transgenic mice prevented soluble Aβ-induced dendritic spine loss [115].

### Summary

Of the two major apolipoproteins found in the CSF, apoE can associate to a number of extracellular molecules and bind to four major CNS apoE receptors; VLDLR, ApoER2, LDLR and LRP. The apoE4 isoform has garnered attention due to the genetic association of apoE4 inheritance and AD risk. ApoE receptors undergo rapid clathrin-mediated endocytosis following ligand binding and have the ability to link ligand binding to several signal transduction pathways. ApoE isoforms exhibit a differential affect on synaptic function and VLDLR and ApoER2 are shown to play a role in synaptic plasticity and memory formation.

### ApoE receptors and AD

**Figure 2:**
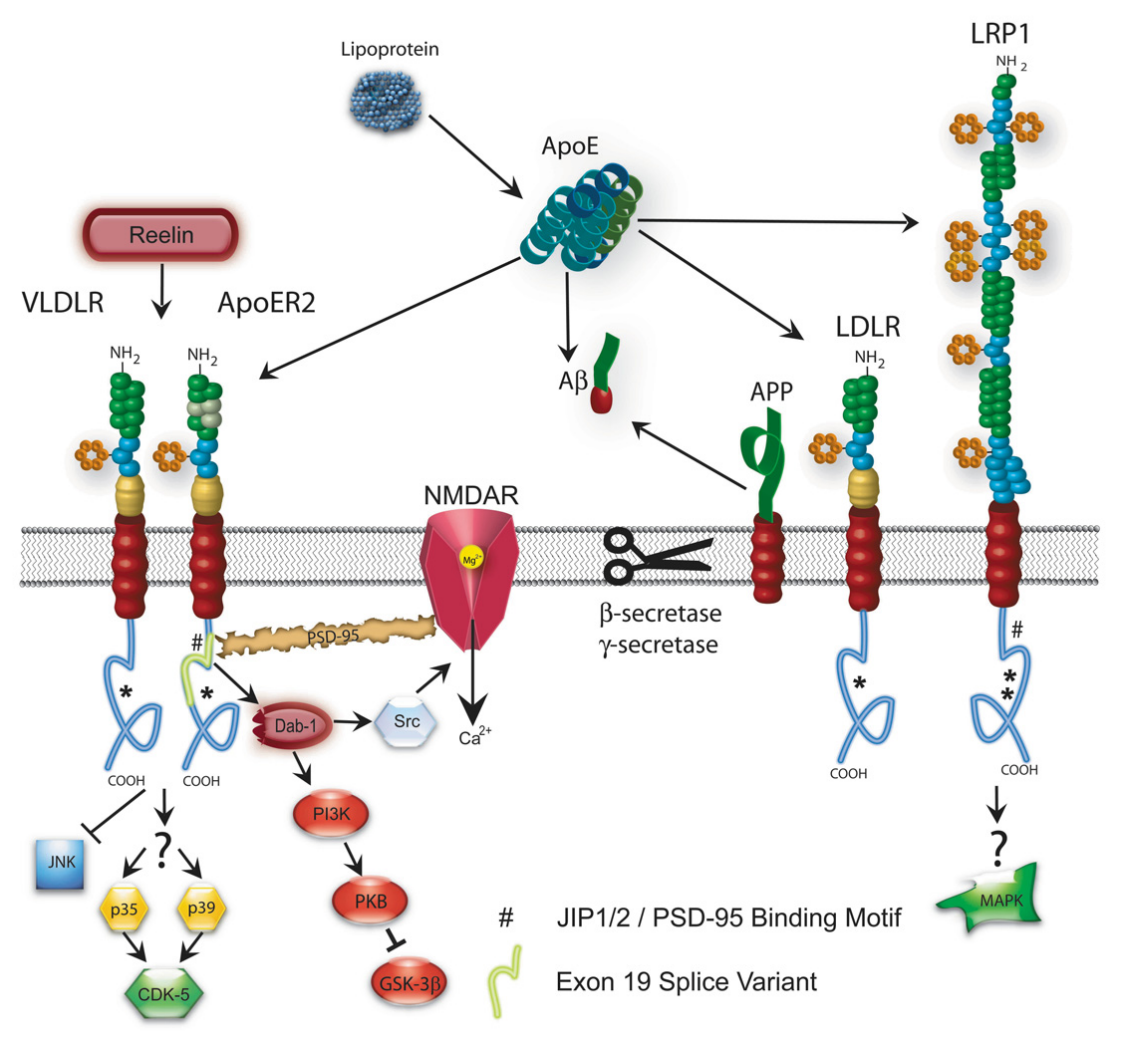
**ApoE receptors and AD pathophysiology**. ApoER2 and LRP receptors can mediate several intracellular signaling kinases including PI3K, CDK-5, JnK and MAPK. The splice variant of ApoER2 expressing exon 19 can influence calcium influx in response to ligand binding by modulating NMDA receptor-mediated currents via a Src-dependent mechanism. Signaling can also be influenced by apoE receptor – APP interaction and specific apoE isoform or Reelin binding.

ApoE receptors are an integral part of normal apoE metabolism, potentially mediating and/or modulating the effects of apoE isoforms on AD pathological processes. They are also important for the cellular homeostasis of cholesterol, which may also affect Aβ production from APP [12]. Several lines of research have implicated apoE receptors directly in AD pathophysiology through several mechanisms (Figure 2).

#### ApoE receptors endocytose Aβ

Almost from the initial observations that apoE bound Aβ [24,34], apoE receptors have been suggested to act as clearance mechanisms for Aβ. Since then, apoE receptors have been found to help transport Aβ across the endothelial cells forming the blood brain barrier [116] or clear Aβ into astrocytes as a degradative process [45]. ApoE is found on most, but not all Aβ deposits in the AD brain [117,118]. LRP is expressed on activated astrocytes [6] and closely associated with Aβ deposits [119]. The importance of Aβ clearance via apoE receptors is also supported by the significant increase in amyloid deposition observed in transgenic APP mice deficient in the RAP gene [120], which has increased levels of several LDLR family members [121,122]. Thus, the interactions of apoE complexes with the apoE receptors in the CNS vitally affect not only the metabolism of apoE, but of Aβ as well.

#### LRP alters APP trafficking and processing

ApoE receptors also have been implicated in the production of Aβ. LRP interacts with APP through the intracellular adaptor protein FE65 or via direct binding to the KPI domain [90,123-126]. Functionally, LRP's rapid endocytosis facilitates APP endocytic trafficking and Aβ production [127-129]. Overexpression of a functional LRP minireceptor *in vivo* resulted in an increase of soluble Aβ in the brain [130].

#### Other members of the LDLR family alter APP trafficking and processing

The apoE receptor LRP1B, which undergoes a slow endocytosis, interacts with APP. However, unlike LRP, expression of LRP1B decreases APP endocytic trafficking and processing to Aβ [131]. ApoER2 also interacts with APP, through an extracellular matrix molecule F-spondin [132] and the intracellular adaptor protein FE65 [78]. These studies suggest that conditions that stabilize APP on the cell surface can increase α-cleavage of APP and decrease Aβ production. Finally, several recent studies have shown that SorLA/LR11 alters APP trafficking to discrete compartments such that APP processing by β/γ-secretases is decreased [133-136]. Together these studies indicate that binding to APP is a common event for the LDLR family members and expression and proteolytic processing of these receptors can impact APP trafficking and processing.

### Summary

ApoE receptors are believed to act as a clearance mechanisms for extracellular Aβ and apoE is often associated with Aβ deposits in post mortem AD brains. The apoE receptors ApoER2, LRP and LRP1B can directly interact with and stabilize amyloid precursor causing increased α-cleavage and reduced Aβ producing cleavage. Thus, apoE and apoE receptors can influence both levels and production of Aβ.

### Soluble apoE receptors

**Figure 3:**
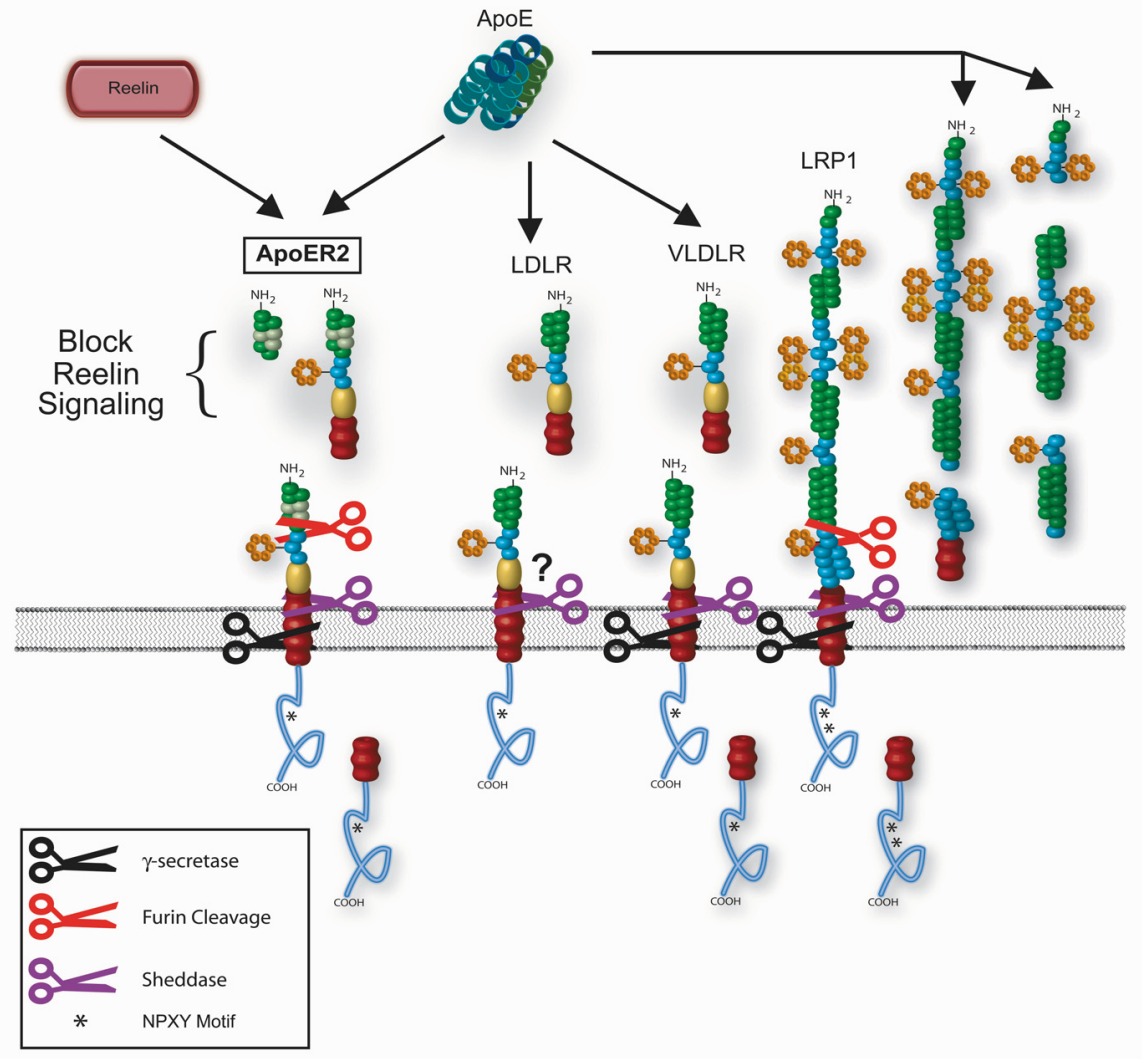
**Soluble apoE receptors**. Processing by γ-secretase cleaves ApoER2, VLDLR, and LRP1 at the membrane. Cleavage by furin can release soluble forms of ApoER2 and LRP. In addition, sheddase cleavage can result in soluble forms of all four receptors. Soluble ApoER2 fragments from furin or sheddase cleavage contain the ligand-binding regions necessary for interaction with Reelin and apoE. These soluble fragments can block endogenous ligand binding to full length receptor acting in a dominant negative fashion.

In addition to the transmembrane forms of apoE receptors, soluble forms of these receptors have been observed *in vitro *and *in vivo *(Figure 3). Soluble receptors can be generated by cleavage of transmembrane forms of the receptors (also called "ectodomain shedding"). Extracellular proteinases responsible for the release of soluble receptors are commonly metalloproteinases, either membrane bound (A Disintegrin and Metalloproteinase, ADAMs) or secreted (Matrix Metalloproteinase, MMPs) [137]. Alternately, soluble receptors can be expressed from alternately spliced mRNAs that lack a transmembrane domain [138]. Both of these processes are important for regulation of soluble apoE receptors and functions of membrane bound apoE receptors.

#### Soluble LRP

LRP is synthesized as a single polypeptide chain of ~600 kDa and then cleaved in the trans-Golgi network by furin into a 515-kDa ligand-binding subunit and an 85-kDa transmembrane subunit that remain non-covalently associated with one another as they traffic to the cell surface [139]. The LRP extracellular region undergoes shedding from a region close to the membrane by metalloproteinases, releasing a soluble LRP (sLRP) capable of binding ligands [140,141]. sLRP is detected in human plasma at nanomolar concentrations [140,141] and in human CSF [142]. Recent studies have also shown that the cell associated fragment of LRP can be cleaved at a third, intramembranous site by the γ-secretase, releasing its intracellular domain (ICD) [143,144]. These sequential cleavage events by furin, metalloproteinases, and γ-secretase closely resemble those of Notch family proteins [145-147].

#### Soluble ApoER2 and VLDLR

Like LRP, ApoER2 and VLDLR undergo extracellular cleavages by metalloproteinases to release soluble receptors as well as C-terminal, cell-associated fragments, and these events are induced by Phorbol esters [148]. Furthermore, the C-terminal fragments are cleaved by γ-secretase [149]. ApoER2 and VLDLR proteolytic events are also increased by extracellular ligand binding [148]. Interestingly, the different apoE alleles induced different degrees of release; both ApoER2 and VLDLR show greatest cleavage following apoE2 activation, less with apoE3 and relatively little with apoE4 [148]. The release of soluble forms of ApoER2 and VLDLR is affected by the presence of splice variants. ApoER2 and VLDLR both have prominent splice variants that lack the exon encoding an O-linked glycosylation site. This region is important in the regulated cleavage of transmembrane proteins [150]. In addition, some ApoER2 splice variants contain an exon that encodes a furin cleavage site in the extracellular domain [59,151]. Furin-dependent cleavage results in extrusion of a soluble fragment of the receptor [152]. Thus, cleavages of ApoER2 and VLDLR are regulated in part by alternate splicing events.

#### Soluble LDLR

Like the other family members, LDLR exists as a soluble form [153,154]. LDLR shedding from the cell surface is enhanced by several stimuli, including interferon and phorbol ester. As for the other apoE receptors, this effect is dependent upon a cell surface metalloproteinase [154]. Inefficient LDLR exon splicing may also contribute to soluble LDLR isoforms because LDLR ESTs have been reported which lack (i) exon 12, which causes a frameshift in the extracellular domain, resulting in a premature termination codon in exon 13, or (ii) exon 15, which encodes the LDLR O-linked glycosylation domain (BG945931 and BQ685399, respectively).

#### Release of soluble receptors

Numerous transmembrane proteins in addition to the apoE receptors have soluble forms, including growth factors and their receptors, cytokine precursors and receptors, cell adhesion molecules, enzymes, and differentiation factors [155]. To gain insight into the possible mechanisms of soluble apoE receptors, we will briefly consider the functions of released extracellular domains of various other transmembrane proteins.

##### 1. Protein function at a distance from the cell

Certain transmembrane proteins have functions that are inactive as long as the protein is tethered to the cell surface. Upon cleavage, these proteins become active and their activities can be carried out at a distance from the cell. For example, TNF-α is synthesized as a membrane-bound, inactive protein but is activated by cleavage by the metalloproteinase TNF-α converting enzyme (TACE) [156]. Soluble TNF-α can then act as a cytokine in the maintenance of inflammation. Similarly, transforming growth factor-α (TGF-α) has a biologically active form on the cell surface, but its activity is limited to the cell surface. However, upon surface cleavage, an active TGF-α is secreted and acts at a distance [157]. Another example of membrane bound protein cleavage is APP. Isoforms of APP containing the Kunitz proteinase inhibitor (KPI) domain can act as serine proteinase inhibitors at the cell surface but, once released from the cell surface, act as soluble proteinase inhibitors [158].

##### 2. An initial step in cell signaling

Ligand binding to a cell surface receptor can transduce a signal inside the cell through several general mechanisms. One of these involves sequential cleavage of the surface receptor after ligand binding, releasing extracellular (soluble) and intracellular (membrane-bound) domains. A subsequent intramembranous (i.e. γ-secretase) cleavage of the membrane-associated protein then releases the cytoplasmic domain for intracellular effects [159]. Thus, release of the soluble receptor is a required step in the signal transduction pathway. The large number of proteins identified as γ-secretase substrates undergo this series of proteolytic events [160]. One well-studied example of this mechanism is the Notch receptor [161]. Notch is a receptor for cell surface proteins on adjacent cells (Delta, Jagged, etc.). After ligand binding, Notch undergoes sequential cleavages to release of its intracellular domain (NICD), which acts as a transcription factor of genes important to development. No function has been assigned the soluble fragment of Notch generated by the initial cleavage. Another example is the proteolysis of SREBP (sterol responsive element binding protein) at luminal and intramembranous sites [162]. These cleavages result in the release of the cytoplasmic fragments of SREBPs that act as transcriptional activators of specific genes important in cholesterol homeostasis.

##### 3. Inhibition of cell signaling

The soluble form of receptors can bind to soluble ligands and prevent them from interacting with membrane-bound receptors, thus preventing their effects on cells. High affinity receptors for several interleukins (IL-1, IL-4, IL-15) have shed forms that bind the specific interleukins and block their actions on cells [163-165]. A soluble version of the receptor for advanced glycation end products (RAGE) can block the interactions of ligands with the membrane bound forms of RAGE, preventing their endocytosis [166], and a soluble version of the leptin receptor blocks leptin signaling [167]. Receptor cleavage can also inhibit signaling that is already occurring on the cell surface. For example, cell surface ephrins interact with Eph receptors on adjacent cells, promoting both forward and reverse cell signaling cascades important in development [168]. The formation of the ligand receptor complex promotes cleavage of the ephrin from the cell surface by ADAMs, thus ceasing both forward and reverse signaling events [169].

##### 4. Release of cell adhesion

Transmembrane proteins are vital for establishing stable connections of a cell with adjacent cells or with the extracellular matrix. When proteolysis of these membrane proteins occurs, the cellular binding to the extracellular matrix is broken, allowing a cell to migrate, or allowing portions of a cell to form new interactions. A component of the cleavage of the ephrin-Eph receptor complex is that this cleavage allows for the induced axonal repulsion [170]. Other examples include the cleavage of cell adhesion molecules L1 that disrupts cell-cell adhesion [171], and cleavage of the discoidan domain receptor 1, that disrupts cell-collagen adhesion [172].

##### 5. Protein turnover

Secreted proteins are often degraded by soluble proteinases, or internalized by cells and degraded in endosomes and lysosomes. Cytoplasmic proteins can be ubiquitinated and degraded by the proteosome [173]. However, transmembrane proteins cannot be sufficiently degraded by either of these mechanisms as long as they are membrane bound. Therefore, turnover of transmembrane proteins requires a combination of proteolytic events. Cleavage at the cell surface would release soluble proteins for extracellular degradation or clearance. Subsequent cleavages within the membrane would generate small protein fragments that could be removed from the membrane, as well as cytoplasmic fragments that could be degraded by intracellular pathways. The urokinase receptor uPAR undergoes a series of cleavages that may be responsible for this type of protein turnover[174]. CD44 is another example of a protein that undergoes these sequential cleavages for degradation [175].

#### Functions of soluble apoE receptors

These potential functions of soluble receptors each apply to apoE receptors. One of the most profound implications of the production of soluble apoE receptors is the possible dominant negative effect on apoE receptor function. This action is observed with the production of soluble ApoER2. The expression of the ApoER2 variant containing the furin consensus site results in the production of soluble receptor consisting of the ligand binding domain [152]. Soluble ApoER2 can effectively block Reelin binding to both ApoER2 and VLDLR and subsequent Reelin-dependent signaling in primary neuronal cells. Thus, inhibition of normal Reelin signaling through ApoER2 and VLDLR can acutely modulate other signaling mechanisms through changes in NMDA receptor activity and intracellular signaling pathways [84-89]. The effect on apoE-dependent signaling has yet to be determined, but these studies suggest that selected apoE receptor shedding would affect both specific ligand binding, as in the case of soluble ApoER2 and Reelin signaling, as well as overall apoE binding and signaling.

Other soluble apoE receptors are shown to act in the same capacity as soluble ApoER2. Soluble LRP can bind RAP in ligand blots [140] and soluble derivatives of LRP, LDLR, and VLDLR have each been shown to mediate receptor-ligand interactions [140,152,176,177]. A physiologic function has yet to be ascribed to the production of soluble apoE receptors. However, in light of the essential roles these receptors play in synaptic function, integration into numerous signal transduction pathways and their wide-range of their ligands, it is likely that this type of negative feedback would be necessary in modulating the activity of specific or multiple apoE receptor subtypes. In addition, cleavage of apoE receptors could be a necessary step in receptor turnover, affecting receptor half-lives, and in preventing uptake of apoE-containing lipoproteins. In summary, release of soluble apoE receptors from the cell surface may modulate the cell surface apoE receptor pathway through multiple mechanisms, including ligand binding away from the cell, altered cell signaling, and differences in receptor degradation.

### Summary

Numerous transmembrane receptors undergo proteolytic processing. Soluble apoE receptors have also been identified for VLDLR, ApoER2, LDLR and LRP. The specific physiologic function for apoE receptor processing has yet to be elucidated. However, a dominant negative effect has been attributed to the processing of ApoER2 and subsequent production of soluble receptor. Thus, specific apoE receptor shedding may represent a novel mechanism for modulating individual apoE receptor-ligand interactions and overall apoE receptor function.

## Conclusion

Soluble apoE receptors are generated by two mechanisms, i.e., proteolysis of transmembrane receptors and by expression of alternately spliced isoforms of the proteins. Moreover, splicing also modulates cell surface proteolysis because exons encoding the O-linked glycosylation domains of VLDLR, ApoER2 and LDLR are alternatively spliced, and this glycosylation domain modulates susceptibility to cell surface proteolysis. These soluble receptors bind their ligands, including apoE, and affect their function and metabolism. The mechanisms and regulation of the processes generating soluble apoE receptors, mediating the actions of these receptors, and controlling the eventual clearance of the soluble receptors are just now being examined. Thus, soluble apoE receptors overall may represent an area of rapid growth in our understanding of AD-related processes. The importance of receptor shedding as a general regulatory mechanism is being recognized in many fields, with shedding of molecules important, for example, in development (Notch, ephrins), immunology (TNF-α, IL-1 receptor, CD44), cell signaling (SREBP, leptin receptors), and cell adhesion (L1, discoidan domain receptor 1). Soluble apoE receptors could play roles as dominant negative regulators of apoE, and thus understanding their generation and actions are important for understanding normal apoE functions in the CNS.
